# Appraisal of Artificial Screening Techniques of Tomato to Accurately Reflect Field Performance of the Late Blight Resistance

**DOI:** 10.1371/journal.pone.0109328

**Published:** 2014-10-03

**Authors:** Marzena Nowakowska, Marcin Nowicki, Urszula Kłosińska, Robert Maciorowski, Elżbieta U. Kozik

**Affiliations:** 1 Department of Genetics, Breeding, and Biotechnology of Vegetable Crops, Research Institute of Horticulture, Skierniewice, Poland; 2 Unit of Economics and Statistics, Research Institute of Horticulture, Skierniewice, Poland; Agriculture and Agri-Food Canada, Canada

## Abstract

Late blight (LB) caused by the oomycete *Phytophthora infestans* continues to thwart global tomato production, while only few resistant cultivars have been introduced locally. In order to gain from the released tomato germplasm with LB resistance, we compared the 5-year field performance of LB resistance in several tomato cultigens, with the results of controlled conditions testing (i.e., detached leaflet/leaf, whole plant). In case of these artificial screening techniques, the effects of plant age and inoculum concentration were additionally considered. In the field trials, LA 1033, L 3707, L 3708 displayed the highest LB resistance, and could be used for cultivar development under Polish conditions. Of the three methods using controlled conditions, the detached leaf and the whole plant tests had the highest correlation with thefield experiments. The plant age effect on LB resistance in tomato reported here, irrespective of the cultigen tested or inoculum concentration used, makes it important to standardize the test parameters when screening for resistance. Our results help show why other reports disagree on LB resistance in tomato.

## Introduction

Tomato (*Solanum lycopersicum* L.) is the fourth most economically important crop in the world: after rice, wheat, and soybean [1], [2]. Of the 200 pathogens affecting tomato production worldwide, *Phytophthora infestans* (Mont.) de Bary, the oomycete causing late blight (LB), is the primary cause of tomato crop loss [2]. Losses are more frequent and more severe in areas where tomato is grown near potato [3].

Research on plant age-dependent expression of *P. infestans* resistance in tomato is needed, especially for the method of inoculation, plant evaluation, and inoculum concentration. In contrast, numerous studies have been done on the methods for testing *P. infestans* resistance in potato [4]–[14]. Tomato germplasm exist with resistance against *P. infestans* [15]–[20]; however, little information is available on standardized methods for evaluating LB resistance in tomato. Information on the correlation between seedlings and mature plant resistance is also limited [21]–[23]. Moreover, the last systematic comparison of methods for testing tomato LB resistance was reported over 40 years ago [24]. Lack of standardization already causes problems. For example, the wild tomato accession *S. pimpinellifolium* L 3708 was reported to have LB resistance conferred by one incompletely dominant gene [15], [25]. That was amended a mere seven years later, when other studies reported complex inheritance of the trait [26]–[29]. Similarly, the accession *S. habrochaites* LA 1033 was designated *Ph - 4* and used as a LB resistance standard by several research centers [30]–[32], despite evidence of multiple QTLs conferring the trait [17], [33]–[35]. Such discrepancies make it difficult for tomato breeders to use resistant germplasms.

The objective of this study was to (i) systematically compare the *P. infestans* resistance in the previously and recently reported resistant tomatoes using an array of methods, both in the field (naturally occurring infections) and with artificial inoculation (employing whole plant and detached leaf/leaflet bio-assays); (ii) determine the importance of the age-dependent expression of LB resistance; and (iii) investigate the level and stability of LB resistance of four sources of this trait in a multi-year field experiment.

## Materials and Methods

### Plant material

Tomato germplasm used in this study included commercial cultivars, breeding lines, landraces, and wild tomato accessions from the tomato germplasm collection at the Research Institute of Horticulture (RIH; Skierniewice, Poland), collectively referred to as ‘cultigens’. Cultigens included three accessions of *S. pimpinellifolium*, five of *S. habrochaites*, three of *S. huaylasense*, two of *S. corneliomuelleri*, three of *S. peruvianum*, and four of *S. lycopersicum*. ‘Rumba’ (*S. lycopersicum*; PNOS Ożarów, Poland) served as the LB susceptible control, and it was readily infected by *P. infestans* under field conditions. The cultigens used in this study, their origin, and LB resistance background (if known) are listed in Table 1.

**Table 1:** Tomato cultigens used in the study, their origin, and LB resistance status (if known).

Cultigen^a^	Origin			Seed source
	country	province	collection site	
***S. pimpinellifolium***				
West Virginia 700 (WVa 700)^b^	-	-	-	INRA, Montfavet, France
L 3707 (PI 365951)^c^	Peru	-	-	Bar-Ilan University, Ramat-Gan, Israel
L 3708 (PI 365957)^d^	Peru	Lima	Pisiquillo	Bar-Ilan University, Ramat-Gan, Israel
***S. habrochaites***				
LA 1033 (6326A)^e^	Peru	Lambayeque	Hacienda Tanlis	NCSU, Raleigh, USA
LA 1353 (365934)	Peru	Cajamarca	Contumasa	TGRC, Davis, USA
LA 2552 (PE 36)	Peru	Cajamarca	Las Flores	TGRC, Davis, USA
LA 2650 (PI 503514)	Peru	Prura	Ayabaca	TGRC, Davis, USA
LA 407	Ecuador			TGRC, Davis, USA
***S. huaylasense***				
LA 1360 (PI 365952)	Peru	Ancash	Apricot	TGRC, Davis, USA
LA 1365 (PI 365953)	Peru	Ancash	Carnaquilloc	TGRC, Davis, USA
LA 1983	Peru	Ancash	Rio Manta	TGRC, Davis, USA
***S. corneliomuelleri***				
LA 1395 (PI 379014)	Peru	Amazonas	Chachapoyas	TGRC, Davis, USA
LA 1910	Peru	Huancavelica	Tambillo	TGRC, Davis, USA
***S. peruvianum***				
LA 1929	Peru	Ica	La Yapana	TGRC, Davis, USA
LA 2581	Chile	Arica i Parinacota	Chacarilla	TGRC, Davis, USA
LA 2744	Chile	Arica i Parinacota	Sobraya	TGRC, Davis, USA
***S. lycopersicum***				
LA 1673	Peru	Lima	Nana	TGRC, Davis, USA
LA 2416	-	-	-	TGRC, Davis, USA
LA 3845 (NC EBR–5)	-	-	-	NCSU, Raleigh, USA
LA 3846 (NC EBR-6)	-	-	-	NCSU, Raleigh, USA
‘New Yorker’ (‘NY’)^f^	-	-	-	INRA, Montfavet, France
LB susceptible control				
‘Rumba’	-	-	-	PNOS Ożarów, Poland

a Plant introductions numbers (PI or PE) were added where available.

b Source of *Ph-2*; single incomplete-dominant gene mapped to the long arm of chromosome 10 [19], [24], [38].

c Source of race-non-specific LB resistance conferred by two independent genes: A partially-dominant gene and a dominant epistatic gene, both mapped to chromosome 9 [46], [47].

d Source of race-specific LB resistance dubbed *Ph-3*; originally reported as conferred by single incomplete-dominant gene, corrected by subsequent research; resistance conferred by at least two QTLs [15], [25]–[29], [31]–[33], [46], [67].

e Source of polygenic LB resistance, conferred by several QTLs [16], [17], [31]–[35].

f Source of LB resistance dubbed *Ph-1*, conferred by a single completely dominant gene mapped to the distal end of chromosome 7 with morphological markers [36]–[39].

Plants were grown from seeds, transplanted at first true leaf stage into ○/ ∅10 cm plastic pots containing Classman Potgrond substrate (Lasland, Grady, Poland), and placed in a greenhouse. Growing plants were kept at approximately 24/18°C day/night for the tests under controlled conditions, or grown for 4 to 5 weeks in the greenhouse, and then transplanted to the field in the second half of May each year.

### Field evaluations

In 2007 and 2008, all cultigens were evaluated for resistance against *P. infestans* under epiphytotic conditions at the Department of Genetics, Breeding, and Biotechnology experimental field area (RIH, Skierniewice, Poland). Four- to five-week old plants were transplanted to the field in a randomized complete block design, with three replications. Each plot consisted of ten *S. lycopersicum* plants or five plants of the wild tomato cultigens, spaced 50×100 cm (within rows × between rows, respectively). Additionally, plants of the LB susceptible ‘Rumba’ were planted around the border of the experimental field to ensure high and uniform pathogen pressure. We also included a *S. lycopersicum* cv. New Yorker (‘NY’) which carries the LB resistance gene *Ph-1* [36]–[39]. No fungicide control was applied throughout the growing seasons in any of the field trials.

Field plots were inspected weekly throughout the season. When the susceptible control ‘Rumba’ foliage developed maximal area symptomatic for LB in a given year, disease ratings for each cultigen were collected. Observations were made by the same individual throughout the experiment to avoid that source of variation. LB lesions on the leaves and stems of each plant were rated using the modified scale of Zarzycka [40]:

- 1 – infection over 97.1 to 100% of the investigated plant organ
- 2 – infection over 87.1 to 97% of the investigated plant organ
- 3 – infection over 75.1 to 87% of the investigated plant organ
- 4 – infection over 50.1 to 75% of the investigated plant organ
- 5 – infection over 30.1 to 50% of the investigated plant organ
- 6 – infection over 18.1 to 30% of the investigated plant organ
- 7 – infection over 10.1 to 18% of the investigated plant organ
- 8 – infection over 3.1 to 10% of the investigated plant organ
- 9 – no infections or small lesions.

At the beginning of this study, such arithmetically biased methods of LB assessment were employed predominantly [5], [8], [15], [19], [20], [24], [25], [31], [35], and keep on being used until this day [9], [16], [21], [26], although more accurate methods were developed [7], [11], [29].

Subsequently, the disease severity index (DSI) was calculated for each cultigen, respectively, as a mean of ratings for the plants, similar to other studies of this pathosystem [26], [40].

In the initial field screenings performed in 2007 and 2008, four cultigens (WVa 700, L 3708, L 3707, LA 1033) exhibited high *P. infestans* resistance. In order to assess the stability of resistance in these cultigens, tests were conducted in 2009 to 2013, under natural LB infestations. Field trials were run in two locations: RIH, Skierniewice (Central Poland), and Boguchwała (Southern Poland), which are 300 km apart from one another. In these experiments, tomato plants were evaluated for resistance against *P. infestans* as described earlier.

### Pathogen cultures and inoculum preparation

**Figure 1:**
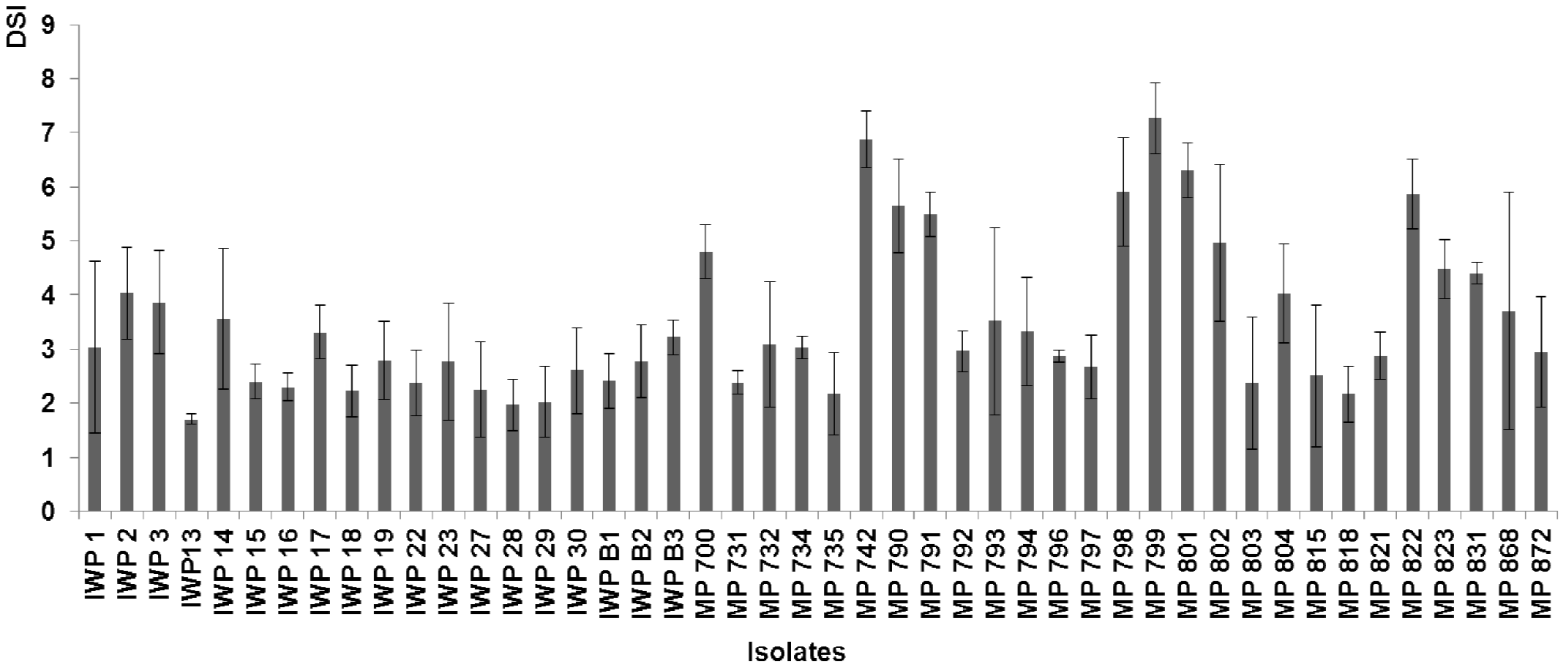
DSI from artificial *P. infestans* inoculations of ‘Rumba’ detached leaflets. A total of 46 local isolates were used. Each isolate was tested on 10 leaflets, in a series of three independent assays. Inoculum concentration of 5×10^4^ sporangia/ml was used. Assay evaluations were performed on the 7^th^ day after inoculation. Disease assessment scale was based on the % of leaflet area being infected; 1 = 100% area infected; 9 =  lack of disease symptoms or few and small necrotic spots. Error bars indicate standard deviation (SD).

*P. infestans* isolates used in this study, collected from tomato plants grown in different regions of Poland and tested in a pilot study (Fig. 1), were deposited at RIH (Skierniewice; n = 19) or obtained from IHAR (the Plant Breeding and Acclimatization Institute - National Research Institute, Młochów, Poland; n = 27 isolates). Isolates were transferred from rye agar onto leaflets of cv. Rumba and cultured for at least two generations, each 7 to 8 days long, with incubations in darkness at 100% RH and 16°C [40]. Inoculum consisted of a sporangial suspension that was washed off the sporulating lesions on the ‘Rumba’ leaflets using distilled water. Sporangia counting was done with a haemocytometer, and final inoculum concentrations (inocula loads) were adjusted according to the assay protocol described below. Prior to dilution, the suspension was chilled for 2 h at 4°C, and then incubated at RT for 30 min.

The isolate IWP 13, collected in 2008 from our experimental field (RIH Skierniewice), was used in all subsequent tests. Detailed isolate characteristics are as follows: race according to Black [3], [4], [7], [10], [11], mating type A1, mtDNA haplotype Ia, and intermediate resistance to metalaxyl [41]. This isolate was chosen based on the results of three independent disease severity tests. These tests were performed with 46 isolates of *P. infestans* on the detached leaflets of cv. Rumba, where it induced the highest disease severity (DSI = 1.7±0.1). Moreover, it produced the smallest variation in symptoms among the isolates tested in a pilot study on ‘Rumba’ (Fig. 1).

### Seedling tests

Resistance screening experiments under controlled conditions were conducted in growth chambers and in greenhouse at the RIH, Skierniewice, Poland. These assays included detached leaflet, detached leaf, and whole plant bio-assays, and are described in detail in the paragraphs that follow. In all test methods we evaluated the influence of plant developmental stage (age in weeks) and inoculum concentration on the disease severity in four tomato cultigens with various levels of *P. infestans* resistance/susceptibility: WVa 700, L 3708, LA 1033, and ‘Rumba’. All tests were conducted in the spring and summer (April to September) of 2011 to 2013.

### The detached leaflet assay

We determined the effects of the plant age (4- to 8-week old plant, in one week increments) and the inoculum concentration (5×10^3^, 10^4^, 5×10^4^ sporangia/ml) on disease symptoms development. Each treatment combination (inoculum concentration vs. plant age) was tested in a series of three independent trials with 25 leaflets per cultigen. The third to fifth fully expanded leaves, counting from the plant's top, were collected. This criterion was established by our earlier experiments as well as by previous studies [42] on the relationship between the intensity of LB symptoms and leaf position on plants. Side leaflets were detached with scissors and immediately placed on wet cellulose wadding in a plastic tray. Sporangial suspension (40 µl) was placed on the center of the abaxial side of each leaflet. Trays with leaflets were covered with glass to maintain 90 to 100% RH and incubated at 16°C. After an initial 24 h incubation in the dark, the leaflets were turned abaxial side down and incubated at the same temperature and RH for 6 days under 12 h photoperiod and a light intensity (PPFD) of 650 µmol/m^2^/s. Symptoms of LB were assessed 7 days after inoculation, using the DSI scale described earlier.

In our preliminary tests with 20 local isolates of the pathogen, individual leaflets of the resistant cultigens displayed high variability in LB reaction. Additionally, the size of necrotic lesions did not correspond with the intensity of sporulation. Within susceptible cultigens (i.e., compatible reactions) that was not observed, and in agreement with other studies [43] the sporulation often preceded the necrosis. These differences caused difficulties in classifying cultigens using this method. Therefore, at the time of assessment by the DSI scale described above, the inoculated leaflets were tested for sporulation intensity by shaking off the sporangia (vortexing each sample for 30 sec in 1 ml of water) and counting the sporangia with a haemocytometer.

### The detached leaf assay

The severity of LB symptoms on detached tomato leaves was studied in 11 stages of development (5- to 15-week old plants, in one week increments), using three concentrations of inoculum (5×10^3^, 10^4^, 5×10^4^ sporangia/ml). The third to fifth fully expanded leaves (counting from the plant's top) were excised from greenhouse-grown plants. The petiole of each leaf was immediately placed into distilled water (100 ml) in a plastic container with a ○/ 1 cm hole in the lid center. The leaves in plastic containers were then placed in a plastic box and hand-sprayed with sporangial suspension, until the upper surface of each excised tomato leaf was completely covered. The inoculated leaves were incubated in darkness for 24 h at 18°C, and 95 to 100% RH. Following this initial incubation, samples were then incubated for 6 days under conditions similar to the detached leaflet method. Leaves of each cultigen were evaluated using the scale described earlier (1 = 97.1–100% leaf area covered with lesions or dead; to 9 =  no lesions). For each of the four cultigens undergoing evaluation, and for each treatment combination (inoculum concentration vs. plant age), a different number of leaves (12 to 24) per cultigen was used due to availability of plant materials. In addition, each treatment combination was examined in three independent sets of experiments.

### The whole plant assay

LB severity on whole plants was tested at six developmental stages (3- to 8-weeks of age, in one week increments) of greenhouse-grown plants, using three concentrations of inoculum (5×10^3^, 10^4^, 5×10^4^ sporangia/ml). Plants were hand-sprayed with the sporangial suspension until complete leaf coverage and excess run-off was observed. The inoculated seedlings were incubated in the dark at growth chamber for 24 h, at 16°C and 100% RH. After this initial incubation, the inoculated seedlings were grown at 16°C with 12 h of light. The plants were rated individually seven days after inoculation. The symptomatic area of both leaves and stems were evaluated using the DSI scale described earlier. Each treatment combination (developmental stage vs. inoculum concentration) was repeated three to seven times, with each cultigen represented by 12 to 72 plants depending on the experiment.

### Statistical analyses

Data from all experiments were analyzed by means of the general linear model with year, location, cultigen, plant age, inoculum concentration, and their interaction as the tested variables. Means were separated with the Tukey multiple comparison procedure at significance level of 0.05. Regression and correlation analyses were used to compare results from the different testing methods. All calculations were done with the statistical software STATISTICA 8.0 (StatSoft, Inc. 2009).

## Results

### Field evaluations

Based on the results from the initial field experiments in 2007 and 2008 (Table 2), four tomato cultigens were chosen (LA 1033, L 3708, L 3707, WVa 700) from the original experimental group of 20 cultigens, to verify their *P. infestans* resistance in the field at two separate locations (Skierniewice, Boguchwała).

**Table 2:** Tomato cultigens' responses to *P. infestans* under natural infection in the Skierniewice experimental field.

Cultigen	DSI^a^		Mean
	2007	2008	
WV 700	8.0±0.5	8.3±0.3	8.2
L 3707	8.0±0.4	9.0±0.0	8.5
L 3708	8.1±0.6	9.0±0.0	8.5
LA 1033	8.8±0.1	9.0±0.0	8.9
LA 1353	6.3±1.1	6.9±0.7	6.6
LA 2552	7.0±0.2	5.5±0.3	6.3
LA 2650	5.0±1.1	5.8±0.8	5.4
LA 407	4.7±1.2	5.3±1.1	5.0
LA 1360	3.1±1.2	2.7±1.1	2.9
LA 1365	3.8±1.5	2.8±0.8	3.3
LA 1983	1.9±0.2	1.5±0.2	1.7
LA 1395	2.6±1.2	2.8±0.7	2.7
LA 1910	1.5±0.2	1.6±0.3	1.6
LA 1929	1.4±0.2	1.8±0.2	1.6
LA 2581	1.0±0.0	1.0±0.0	1.0
LA 2744	1.5±0.2	1.4±0.3	1.5
LA 1673	2.6±1.5	3.0±1.1	2.8
LA 2416	2.1±1.0	2.4±0.7	2.3
LA 3845	1.2±0.2	1.1±0.2	1.2
LA 3846	1.0±0.0	1.0±0.0	1.0
‘NY’	2.6±1.1	1.4±0.2	2.0
‘Rumba’	2.1±1.6	1.2±0.2	1.7

a The disease assessment scale is based on the % area of leaf and stem infection (1 = 100% percent area infected; 9 =  lack of symptoms or few small necrotic spots). Disease symptoms were scored yearly, when LB susceptible control ‘Rumba’ turned fully symptomatic for the infection. Data presented is the mean ± SD from DSI recorded on leaves and stems. LSD_0.05_ calculated according to Tukey procedure for comparing the cultigens in each year: 1.59, and for comparing the years for each cultigen: 0.99.

Comparative analysis of the LB resistance levels among the tested tomato cultigens indicated significant differences across the five years of study (2009 to 2013), at both locations (Table 3). The highest and most stable level of LB resistance in the field, comparable with the baseline years 2007 and 2008, was found in LA 1033 *S. habrochaites* (DSI ranging from 7.2 to 9.0, depending on the year). Plants of this cultigen were either free from any LB symptoms, or developed only slight infection symptoms (classes 6 to 8). Indeed, this cultigen demonstrated high levels of resistance in the field, even under strong *P. infestans* pressure. For example, in 2011, both locations experienced conditions particularly conducive to LB, as confirmed by disease intensities recorded for both susceptible tested tomato cultigens (‘Rumba’, ‘NY’), while LA 1033 showed no LB symptoms (Table 3). Moreover, LA 1033 plants displayed a moderate infection intensity on leaves and stems only in the first week of September 2012 (DSI = 7.2±0.8) and 2013 (DSI = 7.8±0.7), in the Skierniewice experimental field.

**Table 3:** Late blight symptoms severity on chosen tomato cultigens under natural *P. infestans* field infections.

Year^a^	Cultigen	DSI^b^	
		Skierniewice	Boguchwała
2009	LA 1033	8.9±0.1	8.7±0.4
2010		8.8±0.2	8.8±0.2
2011		9.0±0.0	8.0±1.1
2012		7.2±0.8	9.0±0.0
2013		7.8±0.7	8.2±0.6
2009	WVa 700	8.0±0.4	6.1±0.9
2010		8.4±0.3	5.7±1.0
2011		4.9±1.0	5.0±0.7
2012		8.0±0.4	5.7±0.5
2013		4.5±1.1	4.3±1.3
2009	L 3708	7.9±0.4	7.9±0.3
2010		8.6±0.2	8.5±0.2
2011		8.7±0.0	8.0±0.3
2012		8.3±0.4	8.8±0.2
2013		8.2±0.4	7.9±0.7
2009	L 3707	7.8±0.4	7.4±0.6
2010		8.5±0.3	7.5±0.8
2011		8.3±0.7	8.0±0.3
2012		8.3±0.2	8.2±0.2
2013		7.4±0.5	8.3±0.7
2009	‘NY’	4.3±1.4	1.0±0.0
2010		2.4±1.2	1.2±0.2
2011		1.0±0.0	1.2±0.2
2012		1.0±0.0	2.6±0.3
2013		1.7±1.3	1.0±0.0
2009	‘Rumba’	3.6±1.4	1.0±0.0
2010		2.7±1.7	1.4±0.2
2011		1.0±0.0	1.1±0.2
2012		1.0±0.0	1.0±0.0
2013		1.8±0.9	1.0±0.0

a Data were collected from 2009 to 2013 in two locations (Skierniewice, Boguchwała).

b The disease assessment scale is based on the % area of leaf and stem infection (1 = 100% percent area infected; 9 = lack of symptoms or few small necrotic spots). Disease symptoms were scored yearly, when LB susceptible control ‘Rumba’ reached maximal intensity of LB symptoms. Data presented is the mean ± SD from DSI recorded on leaves and stems. LSD_0.05_ calculated according to Tukey procedure for comparing cultigens in each year and in either location: 0.76, and for comparing cultigens in each year and between locations: 0.55.

Intensity of LB symptoms on the *S. pimpinellifolium* accessions L 3708 and L 3707 which ranged from DSI = 7.4 to 8.8, was generally comparable with *S. habrochaites* LA 1033, and depended on the year and the location. The WVa 700 plants displayed significant differences in LB intensity levels, depending on the location and the year (Table 3). In Skierniewice, we recorded partial infection of this cultigen in 2011 and 2013 (DSI = 4.9±1.0 and 4.5±1.1, respectively). In the remaining years of the study, the WVa 700 plants exhibited very low LB intensities (DSI = 8.0 to 8.4). In the Boguchwała study, we observed consistently high levels of disease severity in this cultigen in all years of testing (DSI ranging from 4.3 to 6.1). The ‘NY’ (*Ph-1*) developed intensive LB symptoms, at levels comparable with the susceptibility control ‘Rumba’, irrespective of the location or year of study (DSI = 1.0 to 4.3).

### The detached leaflet assay

**Figure 2:**
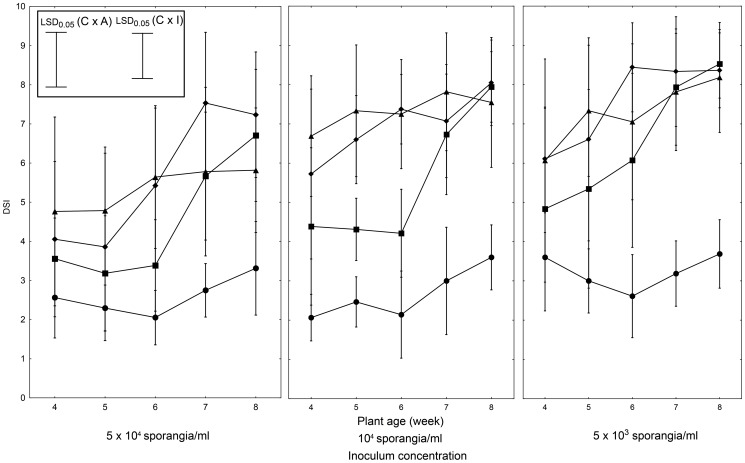
Severity of LB symptoms on tomato cultigens in the detached leaflet assay. Detached leaflets of tomato cultigens (• ‘Rumba’; ▪ WVa 700; ♦ L 3708; ▴ LA 1033) were inoculated with the *P. infestans* isolate IWP 13, at indicated age (weeks), and concentration. Assay evaluations were performed on the 7^th^ day after inoculation. Data shown are the means of ratings from at least three independent experiment sets for each combination; 25 leaflets per cultigen were inoculated per experiment. Vertical bars at each data point signify the standard deviations (SD). LSD_0.05_ calculated according to Tukey procedure (inset) for comparing cultigens at each plant age and inoculum concentration (C×I): 1.34, and for comparing inoculum concentration for each cultigen and plant age (C×A): 1.43.

Experimental results indicated significant effects of the plant age on the LB intensity levels across all tested cultigens. Leaflets from 4- to 5-week old plants of both *S. pimpinellifolium* cultigens tested (WVa 700, L 3708) exhibited higher degrees of infection than those from 7- to 8-week old plants, irrespective of the inoculum concentration used (Fig. 2). The LA 1033 leaflets showed the highest intensity of LB symptoms when inoculated with the 5×10^4^ sporangia/ml (DSI = 4.8 to 5.8, dependent on plant age). Comparatively, at the two lower inoculum concentrations used, the disease symptoms proved significantly decreased at all developmental stages tested.

We found a broad range of intra-cultigen variation in the lesion area of individual leaflets of resistant cultigens (WVa 700, L 3708, LA 1033) inoculated with *P. infestans*. This variation was observed even in the oldest specimens (8-week old) tested with the lowest inoculum load (Fig. 2). The observed high variability of this assay makes it impossible to unequivocally distinguish genotypes exhibiting specific LB reactions. Regardless of the differences in the necrotic area, the LB resistant cultigens showed a low intensity of sporulation and were not significantly different from each other. ‘Rumba’ displayed sporulation intensity of 23,4±5.3 thousands of sporangia/mm^2^, exceeding those of the resistant cultigens approximately 50-fold (WV 700: 0.7±0.6; L 3708: 0.5±0.5; LA 1033: 0.5±0.5 thousands of sporangia/mm^2^, respectively). Also, only in case of ‘Rumba’, did the size of necrotic spots correspond well with the intensity of sporulation. Finally, in contrast to the resistant cultigens tested, ‘Rumba’ showed consistently high and uniform disease symptoms in all detached leaflet assays (Fig. 2), regardless of plant age or inoculum load.

### The detached leaf assay

Significant effects of all variables tested (cultigen, plant age, and inoculum concentration) on the LB intensity were observed. The highest variation was observed for the interaction between cultigen and plant age, and the lowest was between plant age and inoculum concentration.

**Figure 3:**
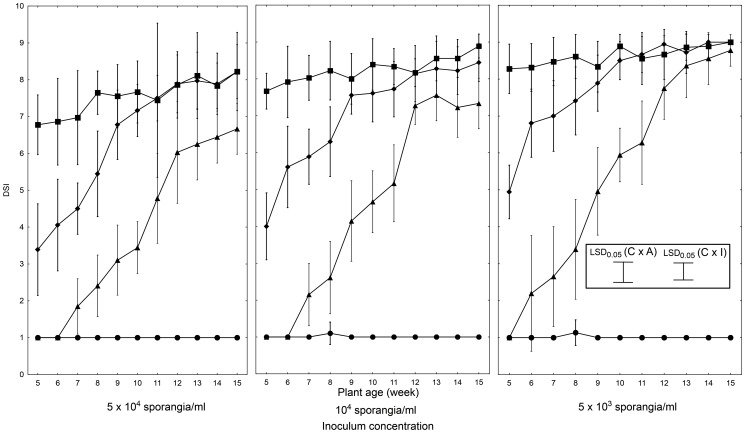
Severity of LB symptoms on tomato cultigens in the detached leaves assay. Third to fifth fully expanded leaves were collected for testing from the plants (• ‘Rumba’; ▪ WVa 700; ♦ L 3708; ▴ LA 1033) at indicated ages (weeks). Detached leaves of the tested tomato cultigens (12 to 24 leaves per cultigen and per developmental stage) were inoculated with suspension of the *P. infestans* isolate IWP 13, by spraying at indicated concentration. Presented data, for each treatment combination, are the means of ratings from three independent experiment sets. Vertical bars at each data point signify the standard deviations (SD). LSD_0.05_ calculated according to Tukey procedure (inset) for comparing cultigens at each plant age and inoculum concentration (C×I): 0.58, and for comparing inoculum concentration for each cultigen and plant age (C×A): 0.74.

‘Rumba’ leaves displayed the highest degree of LB infection (DSI = 1.0 to 1.1), regardless of the plant age, and lacked significant differences in their respective DSI scores (Fig. 3). Relationships between plant age and degree of disease symptoms were observed for all three remaining cultigens in the study (WVa 700, L 3708, LA 1033). Additionally, we noted a different trend in each of the three resistant cultigens. The WVa 700 leaves showed low levels of LB intensity, irrespective of plant age or inoculum concentration. The lowest LB symptoms levels displayed by L 3708 were similar to those of WVa 700, but proved comparatively more dependent on plant age and inoculum concentration (Fig. 3). In L 3708, at the lowest inoculum concentration tested, the impact of plant age on LB intensity was evident, as response dropped below the significance level in 10-week old and older plants. Cultigen LA 1033 also showed varied reactions to *P. infestans* inoculation, dependent on plant age; the observed differences greatly surpassed those noted for WVa 700 or L 3708 (Fig. 3). The lowest degree of disease symptoms in LA 1033 was observed in the leaves of 15-week old plants (DSI = 8.8±0.4), after application of the lowest concentration of inoculum.

### The whole plant assay

As in the detached leaf experiments, also in the whole plant assays we found significant effects of all variables tested (cultigen, plant age, inoculum concentration) on the intensity of LB symptoms. This necessitated an independent analysis for each variable.

**Figure 4:**
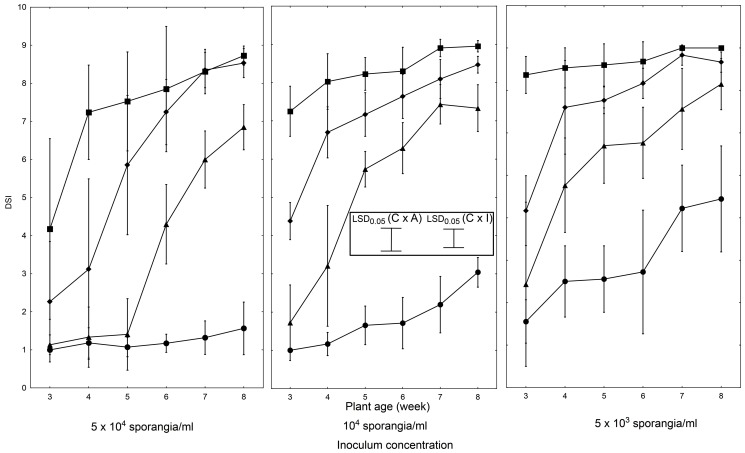
Severity of LB symptoms on tomato cultigens in the whole plant assay. Plants (• ‘Rumba’; ▪ WVa 700; ♦ L 3708; ▴ LA 1033) at indicated ages (3- to 8-weeks) were inoculated with suspension of the *P. infestans* isolate IWP 13, by spraying at indicated concentration (12 to 72 plants per cultigen and per developmental stage). Each combination was tested in three independent experiment sets. Vertical bars at each data point (means of the ratings) signify the standard deviations (SD). LSD_0.05_ calculated according to Tukey procedure (inset) for comparing cultigens at each plant age and inoculum concentration (C×I): 0.55 and for comparing inoculum concentration for each cultigen and plant age (C×A): 0.61.

In ‘Rumba’, we observed a significant interaction between the disease severity and the inoculum concentration (Fig. 4). Plants inoculated with the highest inoculum concentration exhibited high disease severity levels (DSI = 1.0 to 1.6, depending on plant age) in all tested stages of development (3 to 8 weeks of age). In contrast, plants treated with the lowest inoculum concentration showed lower intensity of disease symptoms (DSI = 2.6 to 5.4, depending on plant age). This wide DSI range was dependent on plant age, and for each developmental stage tested was distributed over at least two of the nine severity rating classes (reaching max. 7 classes of spread). A low degree of disease symptoms coupled with large variation in the LB susceptible ‘Rumba’ under 5×10^3^ sporangia/ml, rendered this inoculum concentration unsuitable to correctly differentiate between the LB resistant and LB susceptible plants.

Of the resistant cultigens, WVa 700 had the lowest variation in LB symptoms at different phases of development. Indeed, WVa 700 exhibited a low degree of infection (DSI>7) at all stages of development, regardless of inoculum concentration (Fig. 4), with one exception: the 3-week old plants had increased disease severity with DSI ratings spread over all nine rating classes at the highest inoculum concentration used. We found the lowest, most uniform levels of LB intensity in 7-week old and older WVa 700 plants, regardless of inoculum concentration.

In L 3708, we observed variable levels of LB symptoms intensity, dependent on plant age and inoculum concentration (Fig. 4). Generally, younger plants (3- and 4-week old) exhibited distinctly higher levels of LB sysmptoms than older plants (7- and 8-week old). We also observed higher variation in the range of severity ratings in younger plants compared with older plants. Moreover, the range of variation in severity ratings depended on the inoculum concentration used and correspondingly increased. In addition, we observed an inverse relationship between plant age and inoculum concentration on LB intensity levels. Older plants showed a more uniform intensity of disease symptoms across inoculum loads tested, than did the younger plants. Indeed, among the L 3708 developmental stages tested, the lowest and most uniform levels of LB symptoms were observed in 7- and 8-week old plants at all inoculum concentrations tested.

LA 1033 displayed a more diverse response to *P. infestans* challenges than WVa 700 or L 3708 plants. This reaction, however, remained dependent on plant age at all tested inoculum concentrations (Fig. 4). A comparative analysis of DSI values for all inoculum concentrations at all developmental stages examined in this cultigen showed greater severity of LB symptoms, compared with WVa 700 or L 3708. The lowest level of LB intensity (DSI = 8.2±0.6) was found in 8-week old plants inoculated with the lowest concentration of inoculum. Higher inoculum concentrations caused an increase in the severity of symptoms, with only sporadic significant increases in the variation of severity ratings (Fig. 4).

In general, all control conditions assays indicated an impact of plant age on the intensity of LB symptoms at levels specific for a given tomato cultigen. Symptoms of LB tended to decrease with plant age. High variability noted for the detached leaflet assay makes this method unreliable for standard testing of the LB resistance, or requires additional assessments (e.g., sporulation intensity), but the assays may be used for efficient identification of the LB susceptible individuals, such as during the early stages of selection and breeding.

### Cross-test comparison

**Figure 5:**
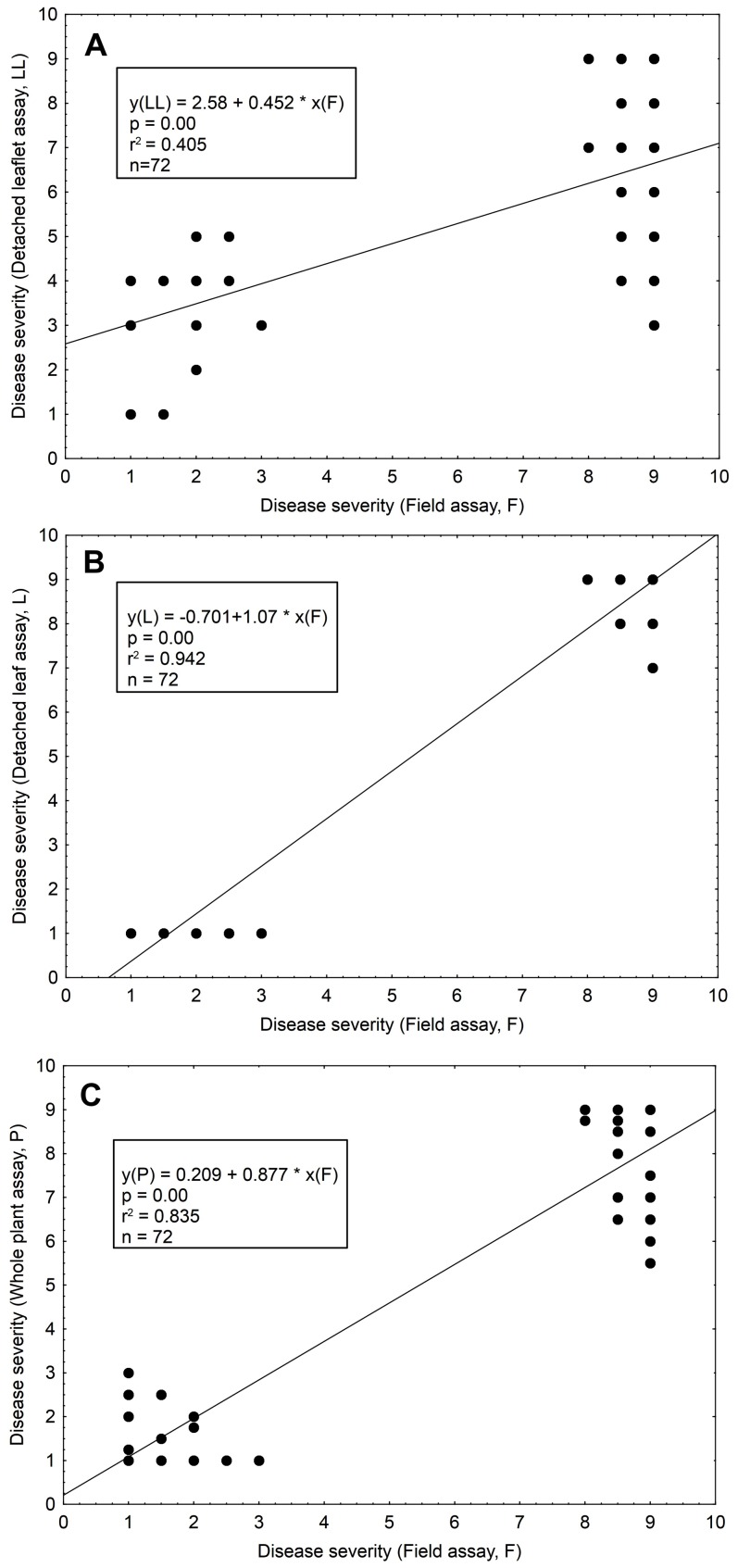
Cross-comparison of the methods for testing the tomato LB resistance. Data from the field experiments (F) were pairwise compared with the results of each of the controlled-conditions method used, under the conditions optimized towards the maximal LB resistance expression (LL: Detached leaflets tested on 8-week old plants, under 5×10^4^ sporangia/ml; L: Detached leaves tested on 15-week old plants, under 5×10^3^ sporangia/ml; P: Whole plants tested on 8-week old plants, under 5×10^4^ sporangia/ml). Calculated trend lines, with respective determination coefficients (r^2^) and *P*-values are indicated. A: Comparison of detached leaflet assays with field assays (LL and F); B: Comparison of detached leaf assays with field assays (L and F); C: Comparison of whole plant assays with field assays (P and F).

Pooled data from the controlled condition tests (detached leaflet, detached leaf, whole plant) from all treatment combinations (cultigens, inoculum concentrations, plant ages), were individually compared with the data of field experiments. All tested laboratory techniques showed significant linear relationships with the field results. But, the detached leaf assay and whole plant assay correlated better with the field assay than did the detached leaflet assay. The determination coefficients were: 0.94, 0.83, and 0.41, respectively (Fig. 5). The stronger relationship observed for the detached leaf assay and whole plant assay with reference to the field data resulted from lower variation within the plant materials, and the domination of extreme DSI values from the field observations.

In summary, this indicates that the detached leaf and the whole plant assays may serve as reliable tools for testing tomato LB resistance. These assays could thus replace the field trials at the early stages of testing. Additionally, it is noteworthy that at high disease intensity (DSI = 1 to 3) the detached leaflet assay showed good correlation with the field results, which further supports our suggestion to use this method for initial testing in tomato LB resistance breeding.

## Discussion

The choice of methods for accurate testing of genotypes for LB resistance is an important area for plant breeding programs, including the tomato-*P. infestans* pathosystem. Lack of agreement among published reports for LB resistance in several tomato cultigens (e.g., L 3708, LA 1033) prompted the current study. Here, our aim was to standardize and compare several methods for testing the *P. infestans* resistance under controlled conditions using four tomato cultigens, over several plant ages and inoculum concentrations. This would allow us to establish a uniform approach to assess the benefit of each cultigen for use in breeding for LB resistance. We also attempted to optimize the methods and to relate the results to those from natural field infection experiments.

Results of the multi-year (2008 to 2013) field experiments, from two locations (approx. 300 km apart), show that *S. habrochaites* LA 1033 had the lowest and most stable *P. infestans* infection levels of the cultigens tested. These findings are in agreement with other studies for LA 1033 reporting high resistance under natural field infection against a diverse set of *P. infestans* isolates, in the USA [17], [33], [35]. In other studies, isolates from Taiwan succeeded in infecting LA 1033 [16], [31], [34]. Our results of LA 1033 showing modest LB symptoms in the field could not be confirmed with detached leaf assay using *P. infestans* isolates derived from symptomatic LA 1033 plants (Nowakowska et al., unpublished data). The latter observation is in agreement with potato-LB studies, where *P. infestans* isolates derived from LB-symptomatic potato plants, carrying the *Rpi-phu1* gene conferring high levels of potato LB resistance, and grown in the field [44], failed to induce the disease symptoms in the laboratory [45]. This demonstrates the complexity of the pathosystem under field conditions.

Other tomatoes with outstanding LB resistance in the field, including the *S. pimpinellifolium* cultigens L 3707 and L 3708 [46], [47], performed insignificantly lower than LA 1033. We observed, however, their stable low LB intensities in the field at all study years and in both locations, even with high *P. infestans* incidence. High LB resistance has been reported in these cultigens [15], [46], [47], despite a lack of clear elucidation of the trait's genetic background [3], [26]–[29]. These cultigens have been successfully used as sources for pyramiding *Ph-3* with *Ph-2* LB resistance into new tomato cultivars [48]–[51]. Only few research groups reported rare instances, when high pathogen pressure, under highly conducive conditions, led to overcoming the resistance of L 3708, particularly in controlled condition assays [15], [30], [31], [52].

In contrast to the aforementioned LB resistant cultigens, WVa 700 [19], [38], [53] exhibited varying levels of LB symptoms in the field, depending on the study year and location. These findings indicate the differences in the local pathogen populations. Consequently, such unstable expression of LB resistance in WVa 700 may occur due to a simpler genetic background for this trait, compared with those found in the other resistant cultigens. Similarly to our findings, this cultigen has failed to display stable LB resistance in other studies [19], [30], [31], [54], [55]. Finally, the *Ph-1* gene present in ‘NY’ [36]–[39] provides no reliable protection against *P. infestans* for field tomatoes grown in Poland, as well as in other locations [2], [24], [30], [32], [54], [56], [57].

Collectively, the field studies indicated that under Polish conditions, LA 1033, L 3707, and L 3708 could be considered promising sources for breeding tomato for LB resistance. This has implications for Central Europe, with field production of both tomatoes and potatoes [1].

In the controlled conditions testing methods used, LA 1033 showed the largest variability in age-dependent reaction to *P. infestans* inoculation. Furthermore, in contrast to its consistently superior performance in the field assays, LA 1033 proved inferior to both WVa 700 and L 3708 cultigens in the detached leaf and whole plant tests. This observation underscores that the assays under controlled conditions may differ from the field tests (weather conditions, plant age, heterogenic isolate mixture, constant pathogen pressure, presence of other (a)biotic stresses). These results also suggest that full expression of LB resistance in LA 1033 occurs later than the oldest developmental stages investigated (3- to 8-week old). Our observation of lower LB intensities in the detached leaves of the 13- to 15-week old plants of this cultigen further supports this hypothesis. The abundant growth of this cultigen may pose problems with generation of appropriate plant materials in the greenhouse for large scale bio-assays. This problem, however, can be solved using the detached leaf tests.

In our study, L 3708 showed higher symptoms variability relative to plant age compared with the WVa 700 cultigen. For L 3708, the trait reached stable expression in 7- to 8-week old plants in the whole plant assays, or in 10-week old leaves. This is in contrast to other studies on L 3708 [15], [25], which described high levels of LB resistance when testing only 5-week old plants. Possible reasons for these discordant results include differences in the assumed methodology (isolates used, inoculum preparation and load, conditions of the assays) or in the climatic conditions related to location (e.g., intensity of sun exposure, length of day).

In the detached leaf and whole plant assays, we recorded the lowest variability for WVa 700, with low LB symptoms levels in plants of this cultigen older than 3 weeks. Moderate LB intensities were seen in whole plant assays. Turkensteen [24] reported age-dependent and progressively increasing LB resistance in the detached leaf assays of 6- to 8-week old WVa 700. Similarly, the 6-week old seedlings of this cultigen exhibited high resistance against both pathogen isolates tested by Moreau et al. [19]. In contrast to our results showing high LB infection in 3- to 4-week old WVa 700 plants, under 5×10^4^ sporangia/ml, previous studies reported low LB intensity in this cultigen, under comparable developmental stages and inoculum concentrations [58], [59]. The most likely reasons for the observed discrepancies are the pathogen isolates used or the assumed methodology, including the double isolate activation employed in our study.

Apart from the significant influence of plant developmental stage and inoculum concentration on the intensity of LB symptoms in the detached leaflet assay, we observed that LB intensity decreases with plant age. Variability of lesion size on leaflets observed using this assay, particularly in the resistant cultigens, indicated the need for improved methods for testing LB resistance (e.g., sporulation intensity). Thus, the detached leaflet assays were an inefficient testing method, although it is fast and easy. The detached leaflet assays can be used only for identification of susceptible genotypes in initial stages of breeding. Our results of tomato LB intensities are in line with the studies of LB in potato [5], [9], [60], [61], where reported problems regarded high variability, especially for potato genotypes with moderate resistance. Although several tomato LB studies used the detached leaflet method to evaluate LB intensity [16], [33], [34], [42], [43], [62], they generally included additional evaluations, such as sporulation intensity. In contrast to the detached leaflet assays, other methods used in this study (detached leaf and whole plant assays) proved more reliable for testing tomato LB resistance under controlled conditions. The observed variability among cultigens tested with these methods was significantly lower than this observed for the detached-leaflet assays. These two most effective methods, however, required different inoculum concentrations for successful separation of resistant genotypes. Both detached leaf and whole plant assays suggested an age-related LB intensity. Our results are similar to those using detached leaflet assays, except for more accurate distinction of the resistant genotypes.

Inoculation and incubation conditions influenced the reproducibility of our results. Here, infection and subsequent development of LB symptoms clearly depended on changes in temperature or RH, in accordance with the biology of the pathogen [5], [8], [33], [57], [60], [63]. Standardizing the test methods (choice of isolate, preparation of inoculum, concentration of inoculum, and conditions of incubation after inoculation) resulted in greater precision in distinguishing LB resistant plants. In the light of our findings, we postulate it very useful to study the plant LB resistance in an age-dependent manner and under the controlled conditions, for each cultigen being reported, in order to better reflect field performance. This might provide an explanation for the differences in mapping of the genes or QTLs controlling the LB resistance trait [15], [25], [26], [28], [64]–[67], and might be of help in detailed analyses of the emerging cultigens reported as LB resistant [16], [18], [20], [34], [50].

Of the methods studied, the detached leaf and the whole plant tests resulted in the lowest discrepancies, when compared with the field experiments. This may be due to inoculation of larger plant surface area, which may generate lower assessment errors and permit a more accurate evaluation of LB resistance. Both leaf and leaflet assays may additionally exhibit reactions to *P. infestans* inoculation different from those of whole plants. These may be due to differences in environmental conditions or influences on the physiological and/or biochemical processes, as a result of detachment from the plant. Thus, we propose the whole plant assays as the most reliable method of testing the tomato LB resistance under controlled conditions. This, in the case of some cultigens (notably LA 1033), may pose other challenges, to be circumvented by using alternative methods, such as the detached leaf assays. We agree with previous reports on potato-LB [4]–[12], [14] and tomato-LB pathosystems [21]–[23], that the ultimate assessment of LB resistance should be performed with field tests.

## Conclusions

Our five-year study under natural field infection, in two distinct Polish locations, confirmed low and stable levels of LB symptoms in LA 1033, L 3708, and L 3707 tomato cultigens. These cultigens are useful for resistance breeding programs for Central Europe. Based on field results, we consider the cultigens carrying the *Ph-1* gene (e.g., ‘New Yorker’) an unsuitable source of LB resistance in Poland. The same is true for cultigens carrying *Ph-2* (e.g., WVa 700) if they are used as the only source of LB resistance. Our comparison of three methods for assessing tomato resistance against *P. infestans* under controlled conditions indicated that each method may be used for different purposes in the resistance breeding. The detached leaflet assay proved useful only to separate the LB susceptible genotypes (such as within the segregating populations) and to maintain the pathogen isolates, but was unreliable for systematic screens due to high variability. Tests on detached leaves and whole plants (greenhouse) generated lower variability than those performed on the detached leaflets and were also found to be highly correlated with field tests. Our results indicate congruent trends for age-dependent expression of LB resistance in all tested tomato cultigens, irrespective of the testing method. The plant age-related LB resistance in tomato reported here, shows the need to optimize and standardize the testing parameters when reporting new sources of resistance. As documented in this study, the reliable comprehensive evaluation of a given cultigen, by means of the optimized and well-suited assays, remains crucial to maximize the benefits from the best performing crops.
